# Survival of a Patient with an Esophagopericardial Fistula After Catheter Ablation for Atrial Fibrillation: A Case Report and Literature Review

**DOI:** 10.19102/icrm.2020.110503

**Published:** 2020-05-15

**Authors:** Granit Veseli, Sei Iwai, Jason T. Jacobson

**Affiliations:** ^1^Westchester Medical Center, New York Medical College, Valhalla, NY, USA

**Keywords:** Atrial fibrillation, catheter ablation, pericardial–esophageal injury

## Abstract

Esophageal injury leading to esophagopericardial fistula (EPF) or atrioesophageal fistula is a very rare and dreaded complication of catheter ablation for atrial fibrillation that carries a high mortality rate. We present a case of EPF following radiofrequency catheter ablation for atrial fibrillation and an extensive review of the literature regarding catheter ablation–related esophageal injury.

## Introduction

We present the case of a rare, dreaded complication related to catheter ablation for atrial fibrillation (AF): esophageal injury leading to esophagopericardial fistula (EPF). Atrioesophageal fistula (AEF) is estimated to occur in less than 0.25% of AF ablation procedures and is the third most common procedure-related fatal complication in this regard, behind cardiac tamponade and stroke.^1^ Although fistulous communication with the atrium is the most commonly reported type of esophageal fistula (EF), it is presumed that EPF occurs early on in the progression to AEF. Surgical repair is considered the treatment of choice for AEF; however, it is unclear if this is also true for EPF. Herein, we describe the management of a patient with EPF after AF ablation and present a review of the available literature regarding catheter ablation–related esophageal injury.

## Case presentation

A 59-year-old male was referred to the arrhythmia service in July 2017 for symptomatic persistent AF. The patient was initially diagnosed with AF in 2014, when he presented with palpitations. The patient’s AF was initially paroxysmal but progressed to persistent AF in May 2017 and he was started on amiodarone 200 mg twice daily. Outpatient direct-current cardioversion (DCCV) successfully restored sinus rhythm. The patient reported improvement for two days while in sinus rhythm but experienced a recurrence of exertional dyspnea and was found to be back in AF. The echocardiogram result showed moderate to severe left ventricular dysfunction with an ejection fraction of 30% to 35% with global hypokinesis, mild right ventricular dysfunction, and a mildly dilated left atrium (LA), with no significant valvular disease. Prior cardiac catheterization indicated nonobstructive coronary artery disease (CAD).

**Figure 1:**
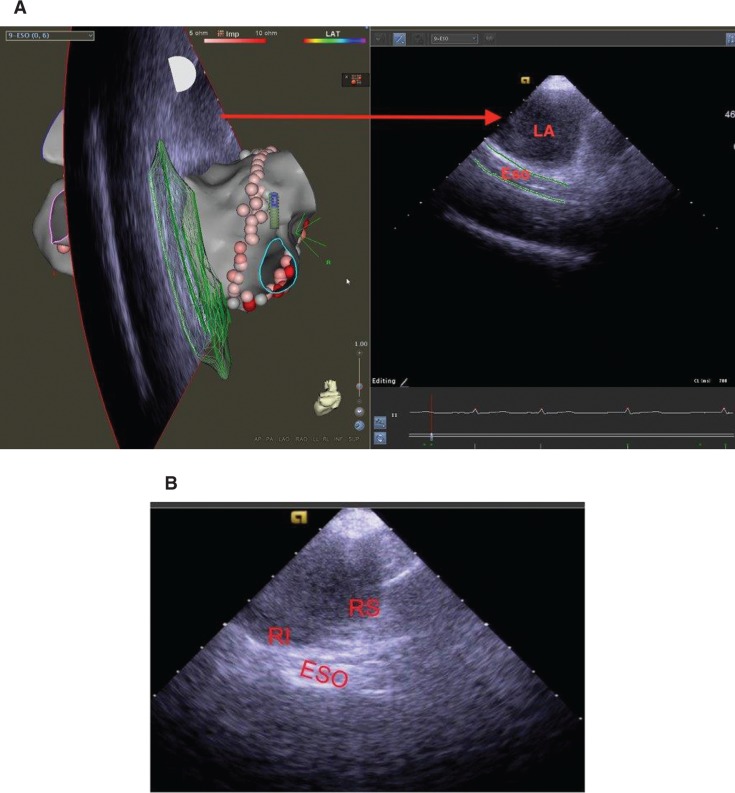
**A:** Left: 3D cardiac anatomic map of the LA posterior wall and esophagus, sourced from the anatomic map and ICE images taken from the right atrium, developed using the CARTO^®^ 3 system (Biosense Webster, Diamond Bar, CA, USA). Right: Image fan from the left panel showing the close apposition of the esophagus (green lines) and LA. **B:** ICE image of the anterior esophageal wall visualized as a hyperechogenic structure running adjacent to the posterior right inferior PV ostium. The right superior PV is further away. Eso: esophageal; RI: right inferior; RS: right superior.

Because of his symptomatic drug-refractory AF, the patient underwent catheter ablation in September 2017, under general anesthesia. A baseline three-dimensional (3D) anatomic map of the LA and esophagus was created with intracardiac echocardiography (ICE) images (CARTOSOUND^®^ module, CARTO^®^ 3 system; Biosense Webster, Diamond Bar, CA, USA) from the right atrium, together with a 3D reconstruction of the cardiac computed tomography (CT) scan; both were incorporated into the anatomic map **(Figure 1A)**. The CARTOSOUND^®^ image shows the anterior esophageal wall, with a hyperechogenic line running in proximity to the right inferior pulmonary vein (PV) **(Figure 1B)**. Wide-area circumferential ablation was conducted with a THERMOCOOL SMARTTOUCH^®^ SF ablation catheter (Biosense Webster, Diamond Bar, CA, USA), with successful isolation of all four PVs. Radiofrequency (RF) energy was delivered with power titrated to a maximum of 30 W while ablating in the anterior portions of the PV antra and to 20 to 25 W while ablating in the posterior wall, with a shorter energy delivery time of 10 seconds to 12 seconds per lesion. Continuous esophageal temperature monitoring was performed with a conventional insulated single sensor catheter during catheter RF energy delivery, which was discontinued if there was any significant or sudden increase seen in the esophageal temperature. Our approach was to discontinue RF energy delivery if the lumenal esophageal temperature (LET) increased more than 0.5°C to 1.0°C as it is expected that the temperature would continue to rise thereafter. Although some operators cease RF energy delivery when the peak LET reaches 38°C to 39°C, we believe that this timing is perhaps too late to prevent thermal injury to the esophagus. Ablation in the affected area was continued after LET recordings showed a decrease in temperature near the baseline, prior to the offending RF lesion. Generally, in the area where the LET increased rapidly, the power was decreased to 15 to 20 W for a shorter duration of 10 seconds. The esophageal thermistor was positioned to the site closest to the planned lesion set and/or the area in closest apposition to the LA wall as identified on ICE.

**Figure 2:**
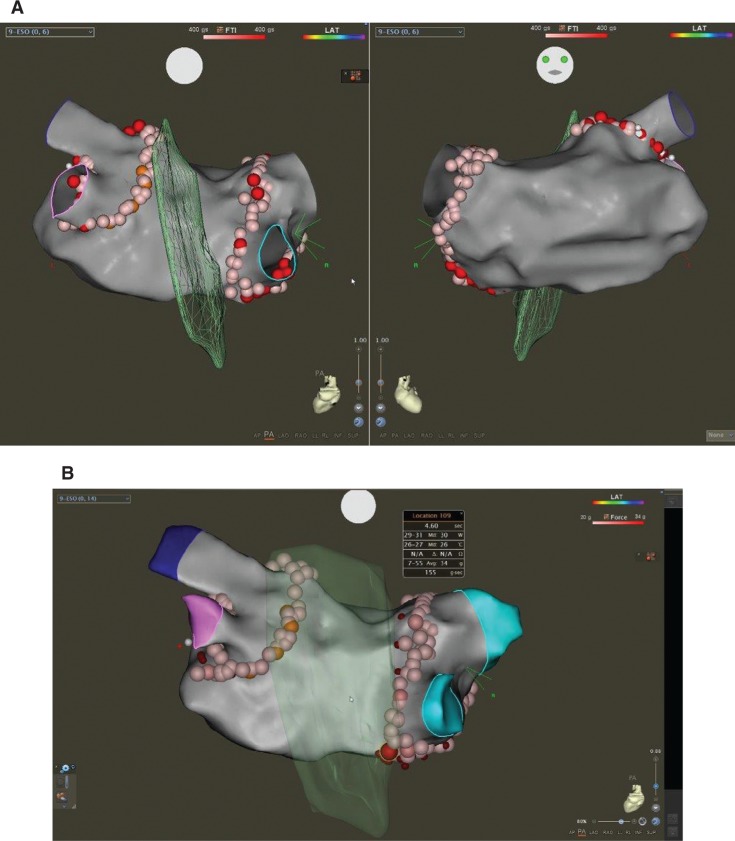
**A:** FTI of the 400-g/s ablation lesions at the antrum of the PVs. Left: posterior view; right: anterior view. **B:** Contact force of the RF ablation lesions on the posterior antral PVs.

When ablating farther from the thermistor position, we tended to be more cautious. Typically, we do not reposition the probe for every lesion as the LET will underestimate the true temperature rise; instead, we cease RF energy delivery with the observation of any subtle temperature increase of more than 0.2°C. Ablation lesions that were created in the posterior wall had an average contact force of 11.9 g. Ablation lesions are displayed based on the force–time integral (FTI) scale up to 400 g/s in **Figure 2A**. Despite careful measures to maintain the contact force around 10 g while ablating in the posterior wall and with an RF energy delivery time of about 10 seconds to 12 seconds per lesion, there was one lesion with a maximum contact force of 34 g implemented for four seconds **(Figure 2B)** at the posterior antral wall of the right inferior PV, with all other lesions in the posterior wall created with a contact force of 20 g or less. Respiratory variation usually leads to intermittent brief intervals of higher contact force during ablation. Pacing was performed from the circumference of both lesion sets and demonstrated a lack of capture. Ultimately, the procedure was completed and was uneventful.

Postoperatively, the patient was started on colchicine and pantoprazole daily for one month’s duration and oral anticoagulation was continued (having previously not been stopped). A few hours after ablation, the patient showed hypotension and decompensated congestive heart failure, but left/right heart catheterization (LHC/RHC) revealed findings of normal cardiac output (9.5 L/min) and cardiac index (5.0 L/min/m^2^) by both Fick and thermodilution methods; further, the mean pulmonary artery (PA) pressure was 25 mmHg, the pulmonary capillary wedge pressure (PCWP) was 12 mmHg, and there was no significant CAD. He underwent DCCV for atypical atrial flutter with rapid ventricular rates (RVRs) and was discharged home in his baseline state of health.

Twenty-four days after AF ablation, the patient presented to the emergency department (ED) of another institution with complaints of lightheadedness and severe chest pain radiating to the back after exercise [ie, picking up a 100-lb (~45-kg) weight]; he had used a stationary bicycle for seven minutes prior to lifting the weight. Upon presentation to the ED, he was afebrile, with normal vital signs and physical examination results, but appeared to be experiencing significant discomfort due to chest pain. A CT scan was performed in the ED, which showed no signs of aortic dissection. The patient subsequently became hypotensive, with a lactic acid level of 13 mg/dL, which was thought to be due to septic shock. He required four pressors (ie, dobutamine, norepinephrine, epinephrine, and vasopressin) for hemodynamic support. A bedside echocardiogram showed a small anterior pericardial effusion and moderate posterior pericardial effusion, but there was no right ventricular collapse. The patient was then transferred for LHC/RHC. A Swan–Ganz catheter and intra-aortic balloon pump (IABP) were placed, with a PA saturation of 74%, cardiac output of more than 7 L, and PCWP of 25 mmHg.

The patient was taken to the operating room for pericardial window and was found to have purulent fluid, with 30 mL drained from the pericardial space and sent for culture. There was no gross evidence of pericardial perforation or LA wall injury per limited views from the pericardial window. A pericardial drain and bilateral pleural drainage tubes were left in place. The patient showed multiorgan failure (manifested by an elevated creatinine level of 4.3 mg/dL, lactate level of 10 mmol/L, aspartate aminotransferase level of 5,878 U/L, and alanine aminotransferase level of 6,008 U/L) despite pressor and IABP support. Hence, extracorporeal membrane oxygenation (ECMO) was initiated; he also required hemodialysis. His presenting rhythm on admission was atypical atrial flutter with RVR (130–140 bpm), and he underwent DCCV to return to sinus rhythm; however, on Day 1 in the hospital, atypical flutter with RVR recurred. Oral amiodarone and metoprolol were started. On Day 2, he had improved hemodynamically, with gradual weaning off of pressors; the ECMO system was explanted on Day 3, and the IABP was removed on Day 5.

**Figure 3:**
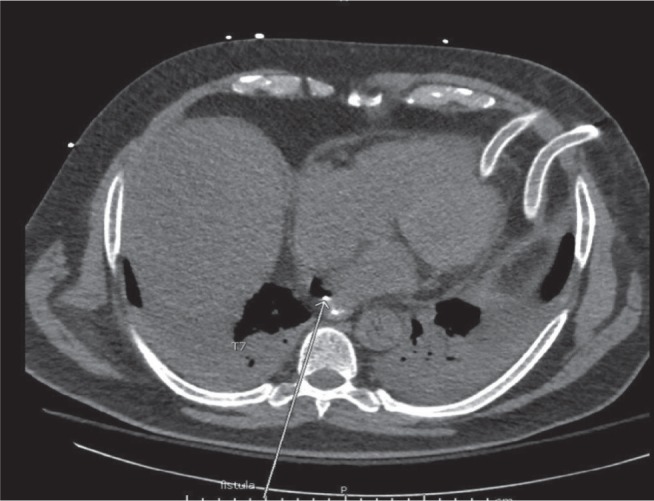
Chest CT scan with oral and intravenous contrast. The arrow indicates the EPF just inferior to the right inferior PV, with extravasation of air and oral contrast into the pericardial space. The left pericardial and pleural drainage tubes are also visible.

**Figure 4:**
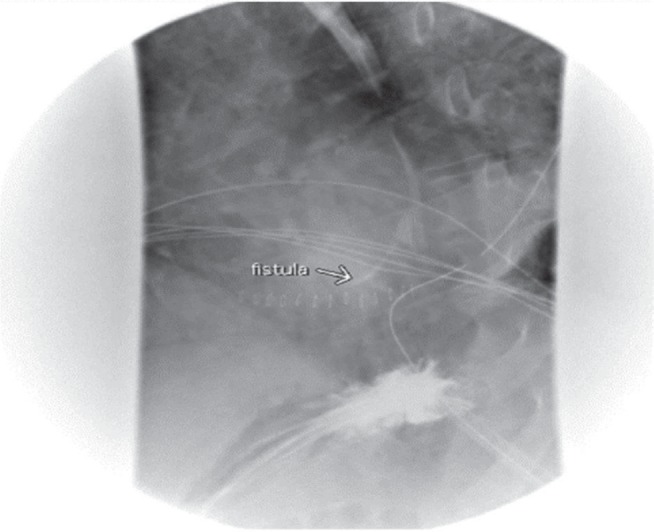
Esophagogram with oral contrast. The arrow indicates the EPF with contrast leak from the esophagus into the pericardial space.

The CT scan with oral contrast (Day 5) showed a loss of the clear fat layer between the posterior pericardium and esophagus just inferior to the right PVs, with extravasation of air and oral contrast from the esophagus into the posterior pericardium, compatible with an EPF **(Figure 3)**. After thoracic surgery and gastroenterology consultation, a decision was made to first place a CORTRACK tube (Avanos, Alpharetta, GA, USA) with the tip at the distal duodenum for postpyloric feeding and to delay placing an esophageal stent for a few days, once sepsis resolved. Repeat esophagram with oral contrast (Day 10) revealed a persistent contrast leak **(Figure 4)**. Therefore, an esophageal stent was placed on Day 12 and a gastrojejunostomy tube was reinserted for postpyloric feeding. A subsequent repeat esophagogram at seven days after stent placement showed no further esophageal contrast leak and stable esophageal diverticulum and, on Day 21, the esophageal stent was removed. Pericardial cultures grew *Streptococcus viridans* and bacillus organisms. The patient completed a three-week course of intravenous ceftriaxone and metronidazole. The patient underwent percutaneous endoscopic gastrostomy (PEG) tube insertion on Day 27. Subsequently, he tolerated a clear liquid diet (Day 32). After 50 days, he was discharged to a rehabilitation facility and continued taking rivaroxaban, metoprolol, furosemide, and amlodipine. Two weeks after discharge, he was advanced to a soft mechanical diet and subsequently to a regular diet three weeks later without issues; thus, the PEG was removed. His functional status progressively improved and his renal function gradually recovered and, at two months after discharge from the hospital, he no longer required dialysis (and his tunneled venous catheter was removed). He was eventually discharged from the rehabilitation facility and returned to normal activities. He also remained in normal sinus rhythm with no evidence of AF by symptoms or mobile cardiac telemetry monitoring.

## Discussion

This case illustrates that early detection of EF, prior to full AEF development, can be managed without esophageal surgery. For a rapid diagnosis, a high level of suspicion and understanding of the time course for EF must be maintained. The first EF complication after catheter ablation for AF was reported back in 2004 and was an AEF.^2^ From a registry study, symptom onset for EF was reported on Day 19 postprocedure (range: 6–59 days). Of the 28 patients with EF, 20 (71%) had an AEF, four (14%) had an EPF, and four (14%) had an esophageal perforation without fistula formation.^3^ Despite adopting caution, this complication is still observed, based on the most recent worldwide assessment, with an esophageal perforation incidence of 0.016% and an AEF incidence of 0.011%.^3^ It is likely that the disease incidence is underestimated since some patients die without a proper diagnosis. Esophageal injury has been shown to occur with the use of all available ablation methods including RF ablation, cryoablation, high-intensity focused ultrasound, robotic RF ablation, and surgical ablation.^4–8^ In a retrospective study of worldwide cryoballoon AF ablation, the incidence of AEF was rare (0.01%) and 90% of AEFs were identified to be near the left inferior PV.^7^

A systematic review of 30 studies that deployed RF catheter ablation as well as cryoablation for AF among 3,921 patients who had undergone endoscopic evaluation of the esophagus within one week after ablation reported that 15% of cases had evidence of esophageal injury. Five patients (0.8%) progressed to esophageal perforation without fistula and four of these five survived following early treatment with an esophageal stent, total parenteral nutrition, and intravenous proton-pump inhibitors and one of the five progressed to EF. Additionally, EF was present in one other (two patients total; 0.3%), who died soon after diagnosis.^9^

The early diagnosis of EF after catheter ablation for AF is essential to maximize the chance of survival. The majority of signs and symptoms of EF are nonspecific and may include fever, fatigue, malaise, chest discomfort, nausea, vomiting, dysphagia, odynophagia, hematemesis, melena, dyspnea, and stroke. Initial laboratory analysis may show signs of infection. The best diagnostic modalities are CT with oral and intravenous contrast or magnetic resonance imaging of the esophagus. Even if an initial CT scan does not show esophageal wall perforation, if clinical suspicion remains high, a repeat CT scan should be completed within a few days. Based on a systematic review, among 11 patients, major abnormalities were detected in 10 (91%) patients on repeat CT chest scans performed six days following the initial scan.^10^ Once an EF diagnosis is suspected, endoscopy is not safe to perform since insufflation can lead to systemic air emboli.

Early intervention is essential, as the mortality rate is 100% without treatment. Various approaches are available for repair, such as endoscopic stenting of the esophagus, primary surgical repair, and placement of a biological barrier and pericardial patching^11^; endoscopic stenting for AEF is far inferior to surgical treatment for mortality reduction.^12^

**Figure 5:**
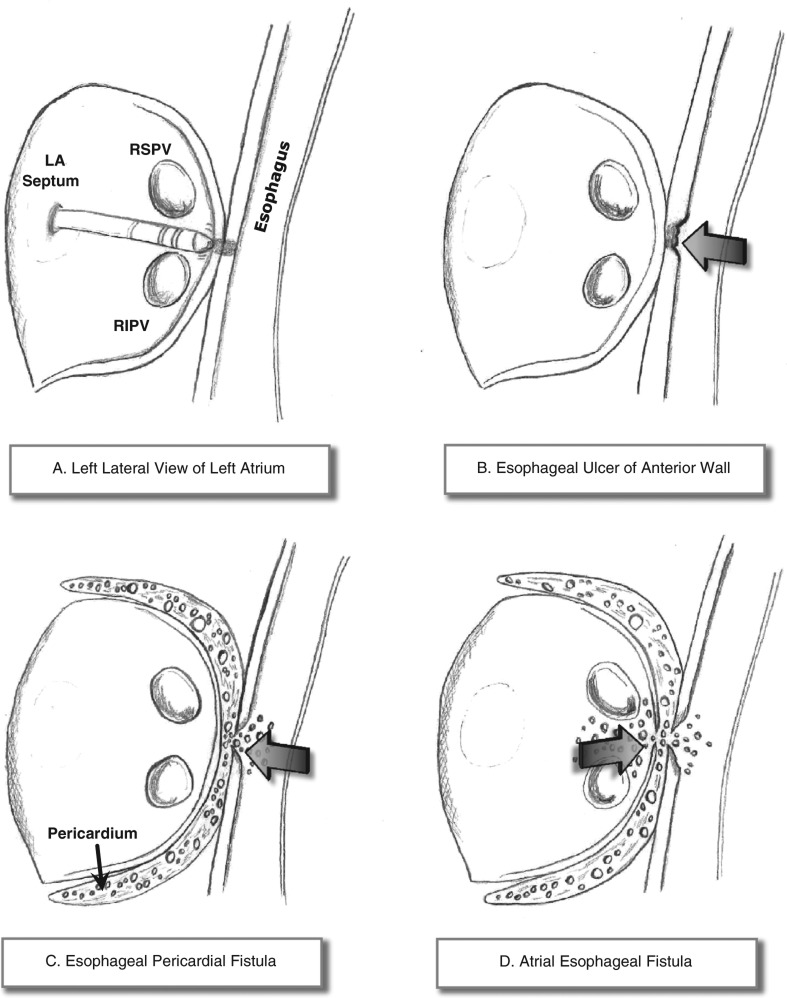
Illustrative figures of esophageal disease spectrum as a complication of AF ablation. **A:** Thermal injury resulting in inflammation and shallow mucosal ulcer at the anterior esophagus wall. **B:** Deep esophageal ulcer. **C:** EPF. **D:** LA–esophageal fistula.

Esophageal injury after catheter ablation for AF can manifest along a pathologic spectrum, ranging from shallow mucosal inflammation to deep esophageal ulceration that can involve the entire thickness of the anterior esophageal wall. This disease can further progress to esophageal perforation that results in mediastinal, pericardial, or atrial fistulization. The spectrum of esophageal injury related to AF ablation is illustrated in **Figure 5**.

There have been only three case reports of EPF, excluding case reports of esophageal perforation with mediastinal fistula.^11,13–15^ Common presenting symptoms of EPF include chest discomfort, fever, and sepsis. All patients survived after prompt diagnosis and treatment with either pericardial drainage and esophageal stenting or surgical repair.

Esophageal injury after AF ablation is not precisely understood; the potential mechanisms of injury include direct thermal injury, acid reflux exacerbation of injury (which can be exacerbated by damage to vagal fibers), infection from the lumen, and microvascular injury.^1,11^ The primary insult appears to be the development of esophageal wall ulceration with eventual fistula formation occurring toward the pericardium and atrium, creating flow from the esophagus to the atrium.^11^ Importantly, patients who had evidence of esophageal ulceration on EGD after ablation were at a significant risk (one in 10) for esophageal perforation.^16^ These data suggest that patients with esophageal ulceration early after ablation require more intense monitoring and, perhaps, more aggressive therapy. However, routine EGD after ablation is not the norm.

Esophageal temperature monitoring is recommended by many operators to help avoid thermal injury. However, there are no data that support this practice; on the contrary, some temperature probes have been shown to increase the risk. For every 1°C increase in esophageal temperature, the odds of an esophageal lesion increase by a factor of 1.36.^17^ One small single-center observational study of 45 patients showed that blocking an absolute-value LET increase of more than 2°C from baseline prevented signs of esophageal injury on follow-up endoscopy. LET was monitored with a 7-French steerable bidirectional 5-mm-tip ablation catheter that was visualized using ICE and moved adjacent to the ablation catheter with each RF application.^18^ Noninsulated temperature probes should be avoided, as they can potentiate heat transfer.^19^ It is worth noting that current probes, including multisensor probes, underrepresent the total esophageal area exposed to thermal injury during ablation. These probes may alter the geometry and position of the esophagus with respect to the LA, potentially increasing the risk of mechanical and thermal damage. Some operators will move the thermistor probe closer to each esophagus-adjacent ablation site to allow for a more accurate representation of LET.^16^ A new technology using infrared thermography offers a more accurate and comprehensive monitoring tool for esophageal temperature monitoring. This probe is a 9-French flexible, radio-opaque catheter capable of scanning 360° and 60 mm of the length of the esophagus. It can obtain 7,680 temperature samples per second.^20^ The temperature sensor continuously moves toward the heat and accurately maps the instantaneous temperature increases in the esophagus. The High-resolution Infrared Thermal Imaging of the Esophagus during AF Ablation as a Predictor of Endoscopically Detected Thermal Lesions (HEAT-AF) prospective trial showed that infrared thermography uses predicted esophageal thermal injury. The mean peak temperature increase was higher (56°C versus 46°C; p < 0.0001) in the group with endoscopic evidence of esophageal thermal injury in comparison with among patients with no evidence of esophageal injury. Based on the HEAT-AF study, binary classification analysis supports that an optimal infrared temperature threshold cutoff of 50°C is likely the best threshold for avoiding thermal injury to the esophagus. Similar results were released by the subsequent AF Thermographic and Endoscopic Monitoring of Patients: Safety Algorithm for the Esophagus (AF TEMP-SAFE) study.^21,22^

The utility of ICE is important in identifying esophageal proximity to ablation target sites. Both real-time esophageal maps using ICE and traditional esophageal maps using esophageal luminal catheters display good correlation in identifying endoluminal borders. However, ICE images are more accurate in identifying the full esophageal anatomy; in 26 patients, ICE-based esophageal borders were located in the area where ablation would have been performed if only traditional imaging was used.^23^ Furthermore, electroanatomic 3D mapping of the esophagus with incorporation into the 3D CARTO^®^ map using the Esophastar (Biosense Webster, Diamond Bar, CA, USA) mapping catheter can guide RF delivery (eg, to deliver lower power for a shorter duration).^24^

New techniques for RF delivery beyond pursuing lower-power/shorter procedural duration are under investigation. One hypothesis is that lower irrigation rates favor transmural lesion creation without endocardial sparing, as seen with fully irrigated RF. In a porcine model, a greater endocardial lesion diameter with less collateral RF tissue injury was seen when limiting the irrigation to 2 mL/min.^25^ The same investigators applied this approach to humans and reported more effective transmural lesions were achieved based on a bipolar electrogram amplitude reduction or elimination of negative deflection in the unipolar distal electrogram and an increase in pacing threshold despite shorter RF time and less energy delivery relative to as seen with high-flow RF lesions.^25^

Alternatively, high-power, short-duration lesions have also been suggested as a means to avoid esophageal heating. In a porcine thigh preparation model, high-power, short-duration RF ablation (50 W applied for five seconds) achieved similar lesion volumes but less lesion depth (2 mm versus 2.9 mm; p < 0.01) when compared with low-power, long-duration lesions (20 W for 30 seconds).^26^ A similar animal study with new ablation catheter technology (Biosense QDot; Biosense Webster, Diamond Bar, CA, USA) demonstrated that high power with a short duration (90 W applied for four seconds with a temperature of less than 65°C) improved RF lesion parameters to yield wider, more contiguous, uniform, and transmural lesions, but the depth achieved was similar to that of standard RF ablation (25 W for 20 seconds).^27^ High power facilitates a larger area of resistive heating, whereas shorter procedural durations result in less conductive heating. Conductive heating extending to deeper tissues may not reach sufficient temperatures and may be one explanation for PV reconnections.^27^ A prospective cohort study at four ablation centers including a total of 10,284 patients undergoing AF ablation with a high-power, short-duration protocol in the posterior LA wall (45–50 W for 2–10 seconds in 11,436 ablations versus 35 W for 20 seconds in 2,538 ablations), illustrated that AEF onset remains very infrequent in the high-power, short-duration population. There was one case of AEF in the high-power group and three cases of AEF in the 35-W group. Of note, two of the three AEF cases occurred in a small subset of 58 patients who did not undergo esophageal temperature monitoring during ablation.^28^

Mechanical esophageal deviation (MED) has been shown to be effective in deviating the esophagus in both directions away from the left and right PV lesion sets, with significant improvements achieved in lowering the increase in LET. The use of a malleable stylet or transesophageal echocardiography probe has been reported for MED, thereby reducing the risk of esophageal thermal injury, but there remains the concern of esophageal trauma stemming directly from the mechanical displacement, which is required multiple times during the ablation.^29,30^ Additionally, an inflatable balloon retractor has been developed for MED. When deflated, the balloon is in a straight position, facilitating easier intubation of the esophagus and, when inflated, the middle segment of the balloon takes a lateral deviation. The use of this device was studied in 200 patients undergoing AF ablation. A deviation of the esophagus of at least 5 mm to 20 mm was achievable in 97.7%. There were no esophageal complications, but two patients experienced oropharyngeal bleeding due to trauma related to device placement.^31^

New strategies for the prevention of esophageal injury exist in early phases of development. An esophageal protective system (EPSac; RossHart Technologies Inc., Cleveland, OH, USA) with cooling circulation and a cooled water–irrigated compliant balloon have shown promise in preclinical testing, although esophageal mechanical expansion from the balloon itself theoretically poses an added risk of thermal injury as the esophageal wall would be compressed against the LA. This method has not gained much popularity among physicians who perform ablation procedures.^32^ More data, including from human studies, are needed to validate the safety and efficacy of such devices.

## Conclusion

The prevention of esophageal injury during posterior LA ablation remains a significant concern, as EF is a potentially life-threatening complication. In this patient, EPF was identified promptly and progression was prevented with esophageal stenting. The challenge remains the creation of a safe and effective method to prevent significant esophageal injury as well as the identification of an optimal technique for monitoring esophageal heating/cooling during ablation.
